# Metabolic associations of human placental lactogen in pregnancies at high metabolic risk: An observational cohort study

**DOI:** 10.1111/aogs.70000

**Published:** 2025-06-25

**Authors:** Kate Rassie, Simon Alesi, Adriana C. H. Neven, Taitum Mason, Eveline Jona, Stacey J. Ellery, Joanne Enticott, Aya Mousa, Anju E. Joham, David Simmons, Helena Teede, TOBOGM Core Investigator Group

**Affiliations:** ^1^ Monash Centre for Health Research and Implementation (MCHRI), Faculty of Medicine, Nursing and Health Sciences, Monash University Melbourne Victoria Australia; ^2^ Department of Diabetes Monash Health Melbourne Victoria Australia; ^3^ The Ritchie Centre, Hudson Institute of Medical Research and Department of Obstetrics and Gynaecology Monash University Melbourne Victoria Australia; ^4^ School of Medicine Western Sydney University Campbelltown New South Wales Australia

**Keywords:** diabetes, fetal macrosomia, gestational, leptin, obesity, placental lactogen, pregnancy

## Abstract

**Introduction:**

Human placental lactogen (hPL) is a placental hormone which, according to preclinical research, appears to have key metabolic roles in pregnancy. We aimed to examine pregnancy hPL levels in relation to maternal metabolic parameters and fetal outcomes within an ethnically diverse cohort at high metabolic risk. Design was an observational cohort study, nested within a randomized controlled trial.

**Material and Methods:**

Pregnant women (*n* = 130), recruited for high metabolic risk, underwent measurement of hPL, plus clinical and metabolic parameters, in early pregnancy (15.8 ± 2.5 weeks of gestation). Univariable and multivariable simple linear regression models were used to examine relationships between early pregnancy hPL and key maternal anthropometric and biochemical variables. Fifty‐four women progressed to serial measurement of hPL and metabolic parameters across pregnancy. Univariable and multivariable mixed effects regression models were used to explore relationships between hPL and maternal variables across pregnancy, with repeated measures adjusted for using random effects.

**Results:**

In early pregnancy, lower hPL levels were independently associated with higher maternal fasting glucose (*β* = −1.03, *p* < 0.01). Early pregnancy hPL was not significantly related to maternal obesity, gestational diabetes mellitus (GDM), or polycystic ovary syndrome status. In women with GDM, sampled serially across pregnancy, maternal hPL and leptin levels were inversely associated (adjusted *β* = −0.098, *p* ≤ 0.001). There was a significant relationship between higher late pregnancy hPL and increased infant birthweight in the serially sampled GDM cohort, both before (*β* = 50.81, *p* = 0.01) and after (*β* = 41.78, *p* = 0.02) adjustment for gestational age at birth.

**Conclusions:**

Maternal hPL may play a role in maternal metabolic adaptation to pregnancy, particularly in relation to glucose and leptin dynamics. hPL in late pregnancy is positively associated with infant birthweight in women with GDM. Future studies of hPL in well‐defined contemporary populations are warranted, both to understand mechanistic interactions in pregnancy and potentially as a biomarker for infant birthweight.

## Graphical abstract

This observational study in an ethnically diverse pregnancy cohort examined relationships between human placental lactogen (hPL), maternal metabolism, and fetal outcomes. Findings support a role for hPL in metabolic adaptation to pregnancy and suggest potential utility as a marker of fetal growth.

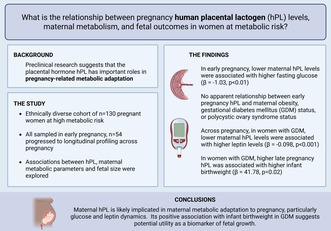

**Abbreviations**
BMI: body mass index
GDM: gestational diabetes mellitus
hPL: human placental lactogen
OGTT: oral glucose tolerance test
PCOS: polycystic ovary syndrome
TOBOGM: Treatment of Booking Gestational Diabetes Mellitus trial

**Key message**

Maternal human placental lactogen is associated with metabolic adaptation to pregnancy and with glucose and leptin dynamics. Late pregnancy human placental lactogen levels are linked to birthweight in pregnancies affected by gestational diabetes, highlighting its potential as a predictive biomarker for macrosomia.

## 1 INTRODUCTION

Human pregnancy is defined by complex and integrated metabolic changes, which are essential to regulate nutrient availability and ensure the health of the mother and the developing fetus. In late gestation, rising maternal insulin resistance ensures the availability of glucose and amino acids for delivery across the placenta.^1^ In order to maintain glucose homeostasis, this is paralleled by a compensatory increase in maternal insulin synthesis and secretion.^2^ The precise endocrine mechanisms underlying these changes, however, remain incompletely understood.

Human placental lactogen (hPL), previously known as human chorionic somatomammotropin, is a polypeptide hormone produced during pregnancy by the syncytiotrophoblast cells of the placenta.^3^ As a member of the somatotropin family, hPL is structurally homologous to pituitary growth hormone (GH), prolactin, and placental growth hormone.^2^, ^3^ In humans, hPL binds mainly to the prolactin receptor, with lower affinity for the GH receptor.^2^ First detected in maternal plasma at approximately six weeks gestation, hPL concentrations increase linearly thereafter until about the 30th week of pregnancy, ultimately reaching peak levels of 5000–7000 ng/mL. The secretion rate of hPL near term is about 1 g per day, a rate considerably greater than that of any other protein hormone.^4^

Along with estrogen, progesterone and prolactin; hPL promotes ductal and alveolar growth in the human mammary gland during the third trimester of pregnancy, in preparation for lactogenesis (hence its nominal designation as a ‘lactogenic’ hormone).^5^ While its role in lactation is well‐established, preclinical evidence suggests that hPL also has important metabolic roles; particularly with respect to carbohydrate and lipid metabolism and altered nutrient availability in pregnancy.^3^ It is likely to contribute to pregnancy‐induced insulin resistance (indeed, most endocrine texts still describe hPL as a ‘diabetogenic’ hormone with insulin antagonistic effects). However, it also has a probable parallel role in maternal pancreatic beta cell adaptation to pregnancy and upregulation of glucose‐stimulated insulin secretion.^2^, ^4^, ^6^ As such, altered hPL dynamics have been implicated in the pathophysiology of gestational metabolic disorders such as gestational diabetes mellitus (GDM); although exact relationships remain unclear^2^, ^3^ and clinical research in this area is methodologically heterogeneous.

Our recent systematic review and meta‐analysis^7^ synthesized the available observational evidence linking hPL to maternal metabolic outcomes in human pregnancy. Results were suggestive of altered hPL dynamics in pregnancies affected by pre‐existing type 1 diabetes (T1DM), but, despite preclinical evidence for physiological roles in both insulin resistance and maternal beta cell adaptation to pregnancy, our meta‐analysis demonstrated no evidence of an association between absolute hPL concentrations and maternal GDM status (either in early or later pregnancy).^7^ Findings regarding the association between hPL and glycemic/ insulin‐related parameters in healthy pregnant women were inconsistent, likely due to variable study methodology. The association between hPL and maternal body mass index (BMI) was also unclear.

Our systematic review findings did suggest that hPL correlates positively with fetal birthweight, and that late pregnancy hPL is elevated in macrosomic pregnancies, particularly in the context of maternal diabetes.^7^, ^8^, ^9^ hPL was widely used clinically in the 1970s‐1980s (prior to the widespread availability of obstetric ultrasound) to assess fetoplacental well‐being in late pregnancy;^10^, ^11^ but has fallen from routine use in recent decades. While recently overlooked as a biomarker for the antenatal prediction of macrosomia (as an adjunct to obstetric ultrasound),^12^ we suggested that hPL may have diagnostic and therapeutic potential if revisited in well‐defined modern cohorts.

In this study, we aim to address evidence gaps highlighted in the systematic review by examining maternal serum hPL levels in a diverse cohort of well‐profiled pregnant women with metabolic risk factors. We explore baseline relationships between hPL and maternal metabolic parameters in early pregnancy in a cross‐sectional cohort, and then (in a serially sampled subset), we examine the metabolic associations of hPL across pregnancy, including the relationship to infant birthweight.

## 2 MATERIAL AND METHODS

### 2.1 Study design and participants

The current study cohort represents a sub‐study of the Treatment of Booking Gestational Diabetes Mellitus (TOBOGM) trial.^13^ TOBOGM was an international multicenter randomized controlled trial comparing immediate treatment of early (<20 weeks) gestational diabetes mellitus (GDM) with standard care (treatment from 24 to 28 weeks). Full study protocol details have been published elsewhere.^13^, ^14^ Recruitment to the TOBOGM study at the Monash Health/ Monash University site in Melbourne, Australia occurred between 4 weeks and 19 weeks +6 days gestation and was based on at least one risk factor for hyperglycemia in pregnancy (previous GDM, BMI >30 kg/m^2^, first degree relative with diabetes, age ≥40 years, previous macrosomia, polycystic ovary syndrome, or non‐European ancestry). Exclusion criteria included established pre‐gestational (type 1 or type 2) diabetes or other active medical disorders that trial investigators considered a contraindication to participation.

The current sub‐study was designed a priori and was included in study design and ethics submissions for the original trial. Overall, 130 participants from the total Monash cohort of 546 participants were selected using purposive sampling methods, capturing all participants reporting a diagnosis of polycystic ovary syndrome (PCOS) (*n* = 63) along with a random sample of those without the condition (*n* = 67). This approach was designed to enrich the sample for PCOS subjects and to allow meaningful comparisons of clinically diverse groups. The resulting sample constituted the baseline cohort (*n* = 130) for the cross‐sectional component of our analysis. A subset of participants (*n* = 54) consented to additional blood tests and were subsequently assessed longitudinally with serial blood samples across pregnancy.

### 2.2 Clinical and anthropometric parameters

Clinical and anthropometric measures for participants in the current analysis were performed at the first TOBOGM study visit (<20 weeks; mean gestation 13.9 ± 2.6 weeks, conducted between January 2018 and December 2019), and/or reported on baseline study questionnaires. Pre‐pregnancy weight, medical history, and PCOS status were self‐reported. A 2‐h 75 g oral glucose tolerance test (OGTT) was performed in all women as part of the main trial at <20 weeks (mean gestation 15.8 ± 2.5 weeks), and women in the current sub‐study consented to have additional fasting bloodwork at the same draw for hPL and additional metabolic parameters.

For the serial testing in the longitudinal component of the sub‐study, 54 participants consented to further blood sampling in mid pregnancy (~24–28 weeks) and again in late pregnancy (~36 weeks) for hPL and additional metabolic parameters. Clinical and anthropometric parameters for these participants were obtained at TOBOGM study visits at corresponding gestations. All 54 of these women had a diagnosis of GDM, either diagnosed on the early OGTT at <20 weeks (*n* = 24) as part of the TOBOGM methodology, or at 24–28 weeks (*n* = 30), consistent with routine care. GDM was defined according to the International Association of the Diabetes and Pregnancy Study Groups (IADPSG) diagnostic criteria.^15^ All women diagnosed with GDM received standard care from the time of diagnosis: education, diet and lifestyle advice, written information, and instructions on how to self‐monitor capillary blood glucose levels. Thresholds for initiation and intensification of pharmacotherapy were as per the previously published TOBOGM trial,^16^ with insulin as first‐line treatment and metformin as a second‐line option in select cases.

### 2.3 Biochemical parameters and laboratory analysis

All venous blood samples were collected following an overnight fast. OGTT samples were analyzed immediately as part of the TOBOGM protocol. Additional samples for metabolic parameters were frozen at ‐80°C until the time of analysis, at which time they were analyzed batch‐wise at the Hudson Institute, Monash University.

Commercial enzyme‐linked immunosorbent assays (ELISA) were used according to manufacturer instructions to measure serum concentrations of hPL (IBL International, Germany) and C‐peptide (Abcam, USA). Insulin and leptin concentrations were measured using a 20‐plex Luminex multi‐analyte kit (R&D Systems, USA). All analyses were performed in duplicate, with the average of measurements used to determine the final concentration. A consistent calibrator sample was included across all plates to account for batch effects and to determine inter‐ and intra‐assay coefficients of variation (CV).

The hPL assay used five standards with a high and low control, and demonstrated intra‐ and inter‐assay CVs of 6.92% and 4.82%, respectively. The C‐peptide assay used six standards, with intra‐ and inter‐assay CVs of 8.48% and 13.41%, respectively. The insulin and leptin analyses, using a 20‐plex Luminex kit with seven standards, demonstrated intra‐ and inter‐assay CVs of 4.8% and 3.8% for insulin, and 4.6% and 2.0% for leptin.

Plasma glucose was measured by Monash Health Pathology using a Hexokinase method on an AU5800 automated chemistry analyzer (Beckman Coulter, Brea, CA, USA). Area under the curve (AUC) for glucose was obtained using the trapezoidal rule.^17^ Homeostatic Model Assessment of Insulin Resistance (HOMA‐IR), a surrogate estimate of hepatic insulin resistance, was calculated as the product of fasting plasma insulin (FPI) and fasting plasma glucose (FPG), divided by the constant 22.5 (ie, FPI [μU/mL] × FPG [mmol/L]/22.5).

### 2.4 Statistical analyses

In determination of outliers, consideration was given to expected trimester‐specific ranges for each value in human pregnancy. Values deemed extreme outliers were excluded from further analysis in order to avoid undue influence on the results of summary statistics and regressions. For two metabolic analytes (insulin and hPL), visual inspection of the distribution of observations at each time point (using histograms and scatter plots) revealed up to four extreme upper outlying values. In the case of insulin, with a normal distribution, the standard rule for determination of outlying values in normally distributed datasets (three standard deviations above the mean)^18^ was applied, resulting in exclusion of two upper outlying values from early pregnancy and one from late pregnancy. For hPL, the distribution of observations at each time point was initially right‐skewed due to a limited number of extreme high values. We identified these outliers using an adapted threshold of 2.5 × the interquartile range above the 75th centile. This approach aligns with the general recommendations for modifying Tukey's fences in the context of skewed datasets.^19^ Following the exclusion of these values (four at early pregnancy, two at mid pregnancy, and four in late pregnancy), the distribution of hPL at each time point approximated normality.

Continuous variables were summarized as means ± standard deviations (SD) if normally distributed or as medians with interquartile ranges (IQR) if non‐normally distributed. Categorical outcomes were reported as counts and percentages. Differences in hPL according to maternal BMI category (obese ≥30 kg/m^2^ vs. non‐obese <30 kg/m^2^), PCOS category, and GDM category were assessed using Wilcoxon rank‐sum tests, given the non‐normal distribution of this parameter.

In the cross‐sectional analysis (*n* = 130) at early pregnancy (<20 weeks gestation), univariable associations between maternal metabolic characteristics and early pregnancy hPL were assessed using simple linear regression, with early pregnancy hPL as the dependent variable. Covariates achieving statistical significance in univariable analysis, and which were deemed potentially relevant based on the existing body of literature,^4^ were selected for entry into the multivariable linear regression model for early pregnancy hPL.

For the longitudinal analysis, mixed effects linear regression models were used to examine associations between human placental lactogen (hPL) levels and metabolic parameters, with hPL as the dependent variable. Univariable models assessed the relationship between hPL, measured at three distinct time points (early, mid, and late pregnancy), and each metabolic parameter individually. Time‐varying predictors (eg, gestational age at visit, systolic and diastolic blood pressure at visit, insulin, C‐peptide, leptin) were modeled as fixed effects, while baseline characteristics (eg, age, parity, weight/ BMI, and oral glucose tolerance test parameters) were included as time‐invariant covariates. Random effects were included to account for participant repeated measures. For the parameters that showed significant univariable associations with hPL, a multivariable model was constructed to assess the independent effects of gestational age at blood draw and leptin on hPL levels.

The relationship between infant birthweight (dependent variable) and hPL at each pregnancy time point (independent variable) was assessed via univariable linear regression, both with and without subsequent adjustment for gestational age at birth.

A significance value of *p* < 0.05 was applied for all statistical tests. Analyses were performed using STATA software version 17.0 (StataCorp LLC, College Station, Texas, USA).

## 3 RESULTS

### 3.1 Sample characteristics

Participants in the baseline cohort, *n* = 130 (Table 1) had an age of 32.9 ± 4.4 years and a baseline (pre‐pregnancy) BMI of 27.7 ± 8.5 kg/m^2^. The cohort was ethnically diverse, with 43.8% of participants identifying as South Asian, 23.1% as White European, and 11.5% as South‐east Asian, with other ethnicities represented in smaller proportions. Overall, 41.5% of participants were nulliparous prior to the index pregnancy. About 18.5% of the cohort (31.5% of the parous women) reported a previous pregnancy affected by GDM, and 48.5% of the cohort reported an existing PCOS diagnosis. Both past GDM and PCOS were high‐risk selection criteria for the study.

**TABLE 1:** Baseline data for whole cohort, first trimester of pregnancy, *n* = 130.

Characteristics, *n* = 130	
Age at first trimester (years)	32.9 ± 4.4
Ethnicity (%)	
White European	30 (23.1)
Middle Eastern	4 (3.1)
South Asian	57 (43.8)
South‐east Asian	15 (11.5)
East Asian	10 (7.7)
Pacific Islander	2 (1.5)
Other	12 (9.2)
Parity prior to index pregnancy	0.8 ± 0.9
Nulliparous prior to index pregnancy (%)	54 (41.5)
Previous GDM (%)	24 (18.5)
Previous impaired glucose tolerance (%)	9 (7.3)
PCOS (%)	63 (48.5)
Family history of diabetes (%)	53 (41.1)
Previous macrosomia (>4000 g) (%)	6 (4.6)
Gestation (weeks) at first clinical visit	13.9 ± 2.6
Gestation (weeks) at blood draw	15.8 ± 2.5
Weight pre‐pregnancy (kg)	73.5 ± 25.3
BMI pre‐pregnancy (kg/m^2^)	27.7 ± 8.5
Weight at first clinical visit (kg)	76.1 ± 25.1
BMI at first clinical visit (kg/m^2^)	28.8 ± 8.4
Systolic blood pressure at first clinical visit (mmHg)	111.1 ± 10.9
Diastolic blood pressure at first clinical visit (mmHg)	67.2 ± 8.9
Fasting glucose (mmol/L)	4.6 ± 0.4
1 h glucose (mmol/L)	8.3 ± 2.1
2 h glucose (mmol/L)	7.1 ± 1.9
AUC glucose	14.1 ± 2.9
Fasting insulin (pmol/L)	38.3 ± 24.9
HOMA‐IR	1.3 ± 0.9
C‐peptide (nmol/L)	0.42 (0.25, 0.60)
Human placental lactogen (mg/L)	2.5 (1.4, 4.0)
Leptin (ng/mL)	9.9 (6.8, 14.9)

*Note*: Continuous variables are presented as mean (±SD) if normally distributed, or median (IQR) if non‐normally distributed. Categorical variables are presented as *n* (%).

Abbreviations: AUC, area under the curve; BMI, body mass index; GDM, gestational diabetes mellitus; HOMA‐IR, Homeostatic Model Assessment of Insulin Resistance; PCOS, polycystic ovary syndrome.

### 3.2 Early pregnancy hPL levels overall and according to maternal metabolic status

Median early pregnancy hPL in the baseline cohort, sampled at 15.8 ± 2.5 weeks of gestation, was 2.5 mg/L (IQR 1.4–4.0, Table 1). Median early pregnancy hPL levels did not differ significantly between women with and without pre‐pregnancy obesity: for maternal BMI <30 kg/m^2^, median hPL was 2.2 mg/L (*n* = 85); compared with 2.6 mg/L (*n* = 30) for maternal BMI ≥30 kg/m^2^, *p* = 0.28. There was no difference in early pregnancy hPL levels by PCOS status (median hPL in PCOS subjects 2.5 mg/L, *n* = 61; vs. 2.2 mg/L in non‐PCOS subjects, *n* = 65), *p* = 0.49. Early pregnancy hPL also did not differ significantly between women who developed GDM during the pregnancy (median hPL 2.2 mg/L, *n* = 67) compared with those who did not (2.5 mg/L, *n* = 59), *p* = 0.67.

### 3.3 Associations between early pregnancy hPL levels and maternal metabolic characteristics

Univariable analysis of associations between early pregnancy hPL (dependent variable) and key maternal characteristics (Table 2) showed a significant positive association between hPL and exact gestational age at the time of blood sampling (*β* = 0.34, *p* < 0.001), consistent with the known steep upward trajectory of hPL across early pregnancy. There was a significant inverse association between early pregnancy hPL levels and fasting glucose levels on OGTT (*β* = −1.03, *p* = 0.003). No relationships were observed between early pregnancy hPL and 1 or 2 h glucose, area under the glucose curve, fasting insulin or C‐peptide, or HOMA‐IR. There was no significant association between early pregnancy hPL and anthropometric measures (maternal age, parity, BMI); nor between hPL and maternal blood pressure, or hPL and leptin. In the multivariable model, the inverse relationship between early pregnancy hPL and fasting glucose levels remained significant after adjustment for gestational age at the time of sampling (*β* = −0.88, *p* = 0.009, Table 2).

**TABLE 2:** Univariable and multivariable associations between early pregnancy human placental lactogen (dependent variable) and early pregnancy clinical/ biochemical maternal characteristics, *n* = 126.

(A) Univariable associations	Univariable human placental lactogen	
	*β*	*p*
Age at first trimester (years)	0.006	0.89
Parity prior to index pregnancy	0.16	0.46
Gestation (weeks) at blood draw	0.34	**<0.001**
Weight pre‐pregnancy (kg)	−0.002	0.79
BMI pre‐pregnancy (kg/m^2^)	0.002	0.94
Weight at first clinical visit (kg)	−0.0004	0.95
BMI at first clinical visit (kg/m^2^)	0.006	0.77
Systolic blood pressure at first clinical visit (mmHg)	0.01	0.54
Diastolic blood pressure at first clinical visit (mmHg)	−0.002	0.94
Fasting glucose (mmol/L)	−1.03	**0.003**
1 h glucose (mmol/L)	0.03	0.67
2 h glucose (mmol/L)	0.09	0.34
AUC glucose	0.02	0.67
Fasting insulin (pmol/L)	0.008	0.26
HOMA‐IR	0.11	0.53
C‐peptide (nmol/L)	0.14	0.77
Leptin (ng/mL)	−0.02	0.32

(B) Multivariable associations	Multivariable human placental lactogen	
	*β*	*p*
Gestation (weeks) at blood draw	0.33	**<0.001**
Fasting glucose (mmol/L)	−0.88	**0.009**

*Note*: Data are presented as *β*‐coefficients with corresponding *p*‐values, representing the relationships with human placental lactogen (A) before adjustment, using univariable simple linear regression, and (B) after adjustment for the other variables in the model/table, using multivariable linear regression. Bold denotes statistical significance at two‐tailed *p* < 0.05.

Abbreviations: AUC, area under the curve; BMI, body mass index; HOMA‐IR, Homeostatic Model Assessment of Insulin Resistance.

### 3.4 Associations between hPL levels and maternal metabolic characteristics across pregnancy

The subset of *n* = 54 women with serial samples provided repeated measures in early, mid, and late pregnancy, with blood collected at mean gestational ages of 16.0 ± 2.5, 25.8 ± 1.4, and 35.9 ± 1.4 weeks, respectively (Table 3). All of these participants had a diagnosis of GDM, as they were recruited into active arms of the ongoing TOBOGM RCT. This subset's characteristics were otherwise broadly similar to those of the total cohort, with comparable maternal pre‐pregnancy BMI (27.0 vs. 27.7 kg/m^2^, *p* = 0.49), early pregnancy BMI (28.1 vs. 28.8 kg/m^2^, *p* = 0.51), and parity (35.2% vs. 41.5% nulliparous before the index pregnancy, *p* = 0.34). Maternal age was slightly higher in the serially sampled group compared to the overall cohort (34.3 vs. 32.9 years, *p* = 0.03), although this difference is unlikely to be clinically meaningful. Of the 54 participants, *n* = 23 had GDM managed with dietary modification alone, *n* = 30 had GDM requiring pharmacotherapy (insulin and/or metformin), and *n* = 1 had GDM with unknown treatment status.

**TABLE 3:** Characteristics across pregnancy for the serially sampled cohort, *n* = 54.

Characteristics	Early pregnancy <20 weeks (*n* = 54)	Mid pregnancy 22–29 weeks (*n* = 54)	Late pregnancy 32–38 weeks (*n* = 54)
Age at first trimester (years)	34.3 ± 4.7	—	—
Parity prior to index pregnancy	0.9 ± 0.9	—	—
Nulliparous prior to index pregnancy (%)	19 (35.2)	—	—
Previous GDM (%)	11 (20.4)	—	—
Previous impaired glucose tolerance (%)	4 (7.7)	—	—
PCOS (%)	13 (24.1)	—	—
Family history of diabetes (%)	23 (43.4)	—	—
Previous macrosomia (%)	1 (1.9)	—	—
Weight pre‐pregnancy (kg)	71.4 ± 22.8	—	—
BMI pre‐pregnancy (kg/m^2^)	27.0 ± 7.6	—	—
Gestation (weeks) at clinical visit	14.2 ± 2.6	25.9 ± 1.6	35.6 ± 1.2
Weight at clinical visit (kg)	74.2 ± 22.5	75.4 ± 15.6	80.5 ± 20.4
BMI at clinical visit (kg/m^2^)	28.1 ± 7.4	28.7 ± 5.3	30.7 ± 6.5
Systolic blood pressure at clinical visit (mmHg)	111.2 ± 9.9	108.4 ± 9.6	113.6 ± 9.8
Diastolic blood pressure at clinical visit (mmHg)	68.6 ± 9.5	67.0 ± 8.1	70.5 ± 8.9
Gestation (weeks) at OGTT	16.0 ± 2.5	—	—
Fasting glucose (mmol/L)	4.8 ± 0.4	—	—
1 h glucose (mmol/L)	9.3 ± 2.1	—	—
2 h glucose (mmol/L)	7.9 ± 1.7	—	—
AUC glucose	15.6 ± 2.7	—	—
HOMA‐IR	1.2 ± 0.8	—	—
Gestation (weeks) at blood draw	16.0 ± 2.5	25.8 ± 1.4	35.9 ± 1.4
Human placental lactogen (mg/L)	2.1 (1.3, 2.9)	5.3 (4.1, 6.6)	8.5 (7.5, 10.6)
Fasting insulin (pmol/L)	30.7 (19.1, 44.4)	37.2 (24.8, 62.4)	31.1 (21.3, 51.5)
C‐peptide (nmol/L)	0.41 (0.27, 0.58)	0.49 (0.34, 0.69)	0.49 (0.37, 0.67)
Leptin (ng/mL)	10.1 (6.5, 14.8)	11.2 (7.0, 15.7)	10.1 (5.5, 15.2)

*Note*: Continuous variables are presented as mean ± SD if normally distributed, or median (IQR) if non‐normally distributed. Categorical variables are presented as *n* (%).

Abbreviations: AUC, area under the curve; BMI, body mass index; GDM, gestational diabetes mellitus; HOMA‐IR, Homeostatic Model Assessment of Insulin Resistance; OGTT, oral glucose tolerance test; PCOS, polycystic ovary syndrome.

In univariable mixed effects linear regression analysis (Table 4), there was a clear positive relationship between maternal hPL levels and gestational age (in weeks) at the time of blood sampling; consistent with the known increase in hPL levels across human pregnancy. An inverse relationship between maternal hPL and leptin levels was observed across pregnancy (*β* = −0.099, *p* = 0.01). This remained significant following adjustment for gestational age in the multivariable model (*β* = −0.098, p = <0.001). hPL across pregnancy did not appear to be related to other serially sampled biochemical parameters (insulin, C‐peptide), to maternal blood pressures across gestation, or to baseline anthropometric or glycemic parameters. Late pregnancy hPL was also not significantly different between participants with diet‐controlled GDM (median hPL 8.0 mg/L, *n* = 21) and those with GDM requiring pharmacotherapy (median hPL 8.9 mg/L, *n* = 28), *p* = 0.10.

**TABLE 4:** Univariable and multivariable mixed effects linear regression model examining associations between hPL across pregnancy (dependent variable) and clinical/biochemical maternal characteristics, *n* = 54 (serially sampled cohort).

(A) Univariable model	*β*	*p*‐value
Age at first trimester (years)	0.041	0.49
Parity prior to index pregnancy	−0.15	0.62
Weight pre‐pregnancy (kg)	−0.015	0.23
BMI pre‐pregnancy (kg/m^2^)	−0.04	0.29
Weight at first clinical visit (kg)	−0.015	0.22
BMI at first clinical visit (kg/m^2^)	−0.04	0.28
Systolic blood pressure at clinical visit (mmHg)	0.022	0.45
Diastolic blood pressure at clinical visit (mmHg)	0.019	0.55
Fasting glucose (mmol/L)	−0.58	0.38
1 h glucose (mmol/L)	0.21	0.11
2 h glucose (mmol/L)	0.26	0.11
AUC glucose	0.17	0.09
HOMA‐IR	−0.071	0.56
Gestation (weeks) at blood draw	0.32	**<0.001**
Fasting insulin (pmol/L)	0.0041	0.67
C‐peptide (nmol/L)	0.018	0.98
Leptin (ug/L)	−0.099	**0.01**

(B) Multivariable model	*β*	*p*‐value
Gestation (weeks) at blood draw	0.32	**<0.001**
Leptin (ug/L)	−0.098	**<0.001**

*Note*: Data are presented as *β*‐coefficients with corresponding *p*‐values. In (A), results represent the relationship between hPL as the dependent variable and each predictor individually. The multivariable model in (B) includes gestational age at the blood draw and leptin as independent variables. Time‐varying predictors (gestational age at visit, systolic and diastolic blood pressure at visit, insulin, C‐peptide, leptin) were measured at three distinct time points: early (visit 1), mid (visit 2), and late (visit 3) pregnancy, as was hPL. Baseline characteristics (eg, age, parity, weight/ BMI, and oral glucose tolerance test parameters) were measured in early pregnancy only. Models accounted for within‐subject correlation by including random intercepts for each participant. Estimates represent fixed effects, with statistical significance defined as two‐tailed *p* < 0.05 (denoted in bold).

Abbreviations: AUC, area under the curve; BMI, body mass index; HOMA‐IR, Homeostatic Model Assessment of Insulin Resistance.

### 3.5 Associations between hPL levels and infant birthweight

In the cross‐sectional cohort (*n* = 130), mean infant birthweight was 3257 ± 521 g and mean gestational age at birth was 38.5 ± 1.9 weeks (*n* = 121 due to missing observations). There was no significant relationship between hPL levels in early pregnancy (independent variable) and ultimate infant birthweight (dependent variable) in the cross‐sectional cohort, either on unadjusted simple linear regression (*β* = 12.05, *p* = 0.63, *n* = 118) or following adjustment for gestational age at delivery (*β* = 15.31, *p* = 0.45).

In the serially sampled subset of *n* = 54 (all with GDM), mean infant birthweight was 3192 ± 481 g and mean gestational age at birth 38.6 ± 1.7 weeks (*n* = 53 due to missing observations). There was no significant difference in mean birthweight between the infants of participants with diet‐controlled GDM and those with GDM requiring pharmacotherapy (3240 ± 413 vs. 3155 ± 531 g, *p* = 0.53). hPL levels in mid pregnancy appeared unrelated to infant birthweight (unadjusted *β* = −51.48, *p* = 0.10, *n* = 51; after adjustment for gestational age at delivery *β* = −29.86, *p* = 0.28). There was, however, a significant positive relationship between late pregnancy maternal hPL levels (measured at 35.9 ± 1.4 weeks) and infant birthweight (unadjusted *β* = 50.81, *p* = 0.01, *n* = 49). This remained significant (*β* = 41.78, *p* = 0.02) following adjustment for gestational age at delivery.

## 4 DISCUSSION

In this study, we demonstrate that early pregnancy levels of hPL, a metabolic hormone, are unrelated to maternal PCOS status, BMI category, or GDM status. Lower hPL, however, appears to be independently associated with higher maternal fasting glucose in early pregnancy. In women with GDM, sampled serially across gestation, maternal hPL and leptin levels appear to be inversely associated; and higher hPL in late pregnancy is associated with higher infant birthweight.

Our recent systematic review^7^ synthesized the current literature on hPL in relation to maternal metabolic outcomes in human pregnancy, identifying likely altered hPL dynamics in pregnancies affected by pre‐gestational T1DM, but (on meta‐analysis) identifying no significant differences in hPL levels between women with GDM and controls, either in early or late pregnancy. The findings of the current study support this conclusion, with hPL levels in early pregnancy similar between women who did and did not go on to develop GDM. As suggested in our systematic review, absolute hPL concentrations—particularly in early pregnancy—seem unlikely to have utility as a GDM risk prediction biomarker.

Our finding (in the cross‐sectional cohort, *n* = 130) of an association between higher maternal hPL levels and lower fasting glucose levels in early pregnancy (*p* < 0.01) is, however, consistent with basic physiological data examining the dynamics of hPL in relation to glucose levels in pregnancy. Gaspard et al.^20^ reported an inverse relationship between blood glucose and hPL levels within healthy pregnant individuals: insulin‐induced hypoglycemia caused a ~ 25% rise in hPL levels (peaking at 30 minutes after insulin administration), whereas rapid intravenous administration of intravenous glucose (bringing blood glucose levels to a mean of 22.2 mmol/L) saw hPL fall over a similar timeframe. Similar results were demonstrated in other dynamic interventional studies in this era,^21^, ^22^ leading to the conclusion that (within a pregnant individual), there is likely to be an inverse relationship between blood glucose and hPL concentrations. Botta et al.^23^ examined hPL in relation to blood glucose levels across gestation in individuals with T1DM (*n* = 15), showing a persistent significant inverse relationship between hPL and maternal blood glucose (both prevailing blood glucose at the exact time of the hPL test, and mean blood glucose that day). In contrast, most other observational studies examining the association between fasting glucose and prevailing hPL levels in women with GDM^8^, ^24^, ^25^, ^26^, ^27^, ^28^ (or controls with normal glucose tolerance)^29^, ^30^ have not shown significant relationships. It should be noted that such studies are methodologically heterogenous, sample sizes are small, and sampling is typically done in later pregnancy (when absolute hPL levels are higher on account of higher placental mass): our study's capture of such parameters in early gestation (mean 15.8 ± 2.5 weeks) was methodologically unique. We did not find clear relationships between hPL and any glycemic parameter other than fasting glucose (1 h or 2 h glucose, fasting insulin or C‐peptide, AUC glucose, or HOMA‐IR), which is largely consistent with the body of existing literature as summarized in our systematic review.^7^

Our data suggest that there is no clear relationship between hPL levels and maternal obesity: no difference was observed between the early pregnancy hPL levels of women with and without obesity, and there was no significant association between hPL and maternal BMI (either in the baseline cohort at early pregnancy or in the longitudinal cohort measured across pregnancy). These results are similar to those reported recently by Al‐Hussein et al.^24^ In contrast, Lin et al.^31^ previously reported a significant inverse relationship between maternal body weight and maternal hPL levels (both measured near term, *n* = 187). One other group has also linked maternal obesity with decreased hPL secretion: Cattini et al.^32^, ^33^ reported lower circulating serum hPL levels in obese pregnant women when compared with their lean counterparts, as well as a >75% decrease in hPL RNA levels in term placental tissue from obese (compared with lean) women. They suggested that pre‐pregnancy maternal obesity may disrupt the chromosomal architecture of the placental hPL gene locus, which was consistent with their findings in a rodent model. However, their human sample sizes were very small (*n* ≤ 10 in each group) and results have not been replicated elsewhere. Overall, there remain an insufficient number of studies focused on hPL in relation to maternal obesity to draw clear conclusions.^3^

Data from our serially sampled cohort (*n* = 54, all affected by GDM) also suggested an inverse relationship between maternal hPL levels and plasma leptin levels across gestation. Leptin is traditionally considered an adipose‐derived hormone, but is also secreted from placental trophoblastic cells during human gestation. It has thus been suggested that placental hormones such as hPL may act via paracrine and/or autocrine mechanisms to regulate placental leptin secretion.^3^ Indeed, in work by Coya et al., in vitro administration of hPL was shown to inhibit leptin release from cultured human placental trophoblast cells,^34^ while incubation with leptin did not modify hPL release under similar circumstances.^35^ Our results, which support an inverse relationship between the two hormones across gestation, are broadly consistent with the dynamics described; although more in‐depth examination of the in vivo relationship between hPL and leptin is clearly required.^3^

In general populations of pregnant women, late pregnancy hPL has been shown to be positively associated with both placental mass^4^ and infant birthweight.^36^, ^37^ Our previous systematic review, which focused on hPL in relation to birthweight specifically in pregnancies affected by maternal diabetes, concluded that similar relationships were likely to apply within this population, although the results of individual studies varied.^7^, ^8^, ^9^, ^25^, ^38^ The findings of the current study are similarly suggestive of a positive association between third‐trimester hPL and infant birthweight, both before and after adjustment for exact gestational age at delivery. Given that macrosomia is associated with adverse fetal and maternal outcomes, accurate antenatal prediction of macrosomia remains an area of key clinical interest. From a technical perspective, hPL can be readily measured in serum using commercially available immunoassays and would be feasible to implement in clinical laboratory settings pending further validation. However, while we briefly examined the relationship between late pregnancy hPL and birthweight, a dedicated analysis specifically modeling hPL as a predictor of fetal size—adjusting for key clinical covariates such as maternal BMI, gestational weight gain, hypertensive disorders of pregnancy, GDM status and treatment modality, and glycemic control—was beyond the scope of this study. Such an investigation would require a larger, well‐powered cohort with more complete data and a specific focus on perinatal outcomes. In the modern era, any proposed biomarker for macrosomia would need to demonstrate predictive value above and beyond that already achievable through established clinical tools such as ultrasound biometry and maternal risk stratification (in populations both with and without maternal diabetes). To date, no biomarker has met that threshold for clinical translation, and further prospective validation of candidates such as hPL will be essential to determine their potential clinical utility. Incorporation of hPL into multivariable risk models for macrosomia may also represent a future avenue of investigation, particularly if shown to offer incremental predictive value alongside existing clinical and sonographic parameters.

Strengths of our analysis include the use of a well‐characterized, ethnically diverse cohort with a high prevalence of metabolic risk factors, the inclusion of early pregnancy hPL (uncommon in previous literature, which focuses predominantly on third‐trimester measurements) and the inclusion of serial analyte measurements across pregnancy for a subset of participants. Limitations include the relatively small sample size (particularly for the serially sampled subset), the self‐reported nature of PCOS status, and the fact that our serially sampled cohort was all affected by GDM (with heterogeneity in pharmacotherapy that could not be meaningfully accounted for in our analyses). Finally, the absence of OGTT data beyond the first trimester also meant that the observed inverse relationship between hPL and fasting glucose was unable to be verified at later pregnancy time points.

## 5 CONCLUSION

Maternal hPL levels in early pregnancy may be inversely associated with fasting glucose, but do not appear to be related to maternal obesity, GDM, or PCOS status. In women with GDM, there appears to be an inverse association between hPL and maternal leptin levels across pregnancy, and a positive association between late pregnancy hPL and infant birthweight. Further clinical research focused on the metabolic associations of hPL in well‐defined contemporary populations is required, given the large body of preclinical evidence supporting its role in maternal metabolic adaptations to pregnancy. The potential utility of hPL as a macrosomia prediction biomarker also warrants exploration.

## AUTHOR CONTRIBUTIONS

KR conceptualized the analysis, with oversight from AM, AEJ, and HT. HT led the overall TOBOGM study at the Monash site, with trial co‐ordination by EJ and clinical support from ACHN. DS was the principal investigator for TOBOGM and approved the Monash sub‐study. Laboratory analysis for the current study was led by SA, with support from TM and oversight from SJE. Statistical analyses were performed by KR, with expert biostatistics support from JE. KR drafted the manuscript, which was reviewed and revised by SA, JE, DS, AM, AEJ, and HT, and approved by all other authors. All authors have contributed to this manuscript in line with the ICMJE criteria for authorship and have read and approved the manuscript for publication.

## FUNDING INFORMATION

This research did not receive any specific grant from any funding agency in the public, commercial, or not‐for‐profit sector. KR is supported by a scholarship from the Australian National Health and Medical Research Council (NHMRC). SA and ACHN are supported by Monash University scholarships. SJE, AM, AEJ, and HT are supported by fellowships from the NHMRC.

## CONFLICT OF INTEREST STATEMENT

All authors report no conflicts of interest.

## ETHICS STATEMENT

The TOBOGM study was registered (ACTRN12616000924459) and had ethical approval from the South Western Sydney Local Health District Human Research Ethics Committee (reference 15/LPOOL/551, approval November 9, 2015), and locally from the Monash Health Research Ethics Committee (reference RES‐17‐0000‐406X, approval April 6, 2016). Full study protocol details have been published elsewhere.^14^ All participants provided informed written consent for participation.

## Supporting information




Data S1.

## Data Availability

A data sharing statement relevant to the broader TOBOGM study (from which all clinical, anthropometric, and OGTT data were sourced) is available in relation to that published study.^13^ Biochemical data for the current sub‐study (hPL levels and metabolic analytes) are not currently publicly available, but anonymized data may be obtained from the corresponding author upon reasonable request. Data access will be granted to qualified researchers for purposes of replicating or extending the study, pending institutional approval where required.
