# Supplementary material for: Metabolic associations of human placental lactogen in pregnancies at high metabolic risk: An observational cohort study
Source: Acta Obstet Gynecol Scand. 2025 Jun 25;104(9):1694–704. doi: 10.1111/aogs.70000 (PMC12393997; doi:10.1111/aogs.70000)
Supplement: Supplementary file 1 — Data S1. [file AOGS-104-1694-s001.docx]

**Supporting Information S1**

TOBOGM Core Investigator Group

The “ToBOGM Core Investigator Group” is made up of the investigators who

developed the ToBOGM protocol, applied for the NHMRC Grant and have remained

actively involved:

Professor David Simmons (Chief Investigator and Campbelltown Hospital PI)

Professor William Hague (Robinson Research Institute, The University of Adelaide)

Professor Helena Teede (Monash University)

Professor Wah Cheung (Westmead Hospital)

Professor Christopher Nolan (Australian National University)

Professor Michael Peek (Australian National University )

Associate Professor Jeff Flack (Bankstown-Lidcombe Hospital)

Professor Mark Mclean (Blacktown/Mt Druitt Hospital)

Associate Professor Vincent Wong (Director, Diabetes Services, Liverpool Hospital)

Associate Professor Emily Hibbert (University of Sydney and Nepean Hospital)
